# Responses to Real-World and Hypothetical Menthol Flavor Bans Among US Young Adults Who Smoke Menthol Cigarettes

**DOI:** 10.1093/ntr/ntad259

**Published:** 2023-12-26

**Authors:** Jamie Tam, Evelyn Jimenez-Mendoza, John Buckell, Jody Sindelar, Rafael Meza

**Affiliations:** Department of Health Policy and Management, Yale School of Public Health, New Haven, CT, USA; Department of Epidemiology, University of Michigan School of Public Health, Ann Arbor, MI, USA; Nuffield Department of Population Health, Health Economics Research Centre, University of Oxford, Oxford, UK; Department of Health Policy and Management, Yale School of Public Health, New Haven, CT, USA; Department of Integrative Oncology, BC Cancer Research Institute, BC Cancer Research Institute, Vancouver, British Columbia, Canada; School of Population and Public Health, University of British Columbia, Vancouver, British Columbia, Canada

## Abstract

**Introduction:**

Menthol cigarette bans have been implemented in some US states and localities, and a federal ban is being proposed by the FDA. This study asks how young adults who use menthol cigarettes respond to changes in menthol cigarette availability.

**Aims and Methods:**

An online survey of young adults ages 18–34 who reported smoking menthol cigarettes on ≥7 of 30 days around Thanksgiving 2019 (*n* = 734), oversampling Massachusetts—the first state with a menthol ban. Participants reported their tobacco use behavior following real-world menthol cigarette bans or predicted their behavior under a hypothetical federal ban.

**Results:**

Most respondents who exclusively smoked versus dual used with e-cigarettes continued smoking/using combustible tobacco following real-world bans (95.3% vs. 86.9%), accessing menthol cigarettes from other jurisdictions. Fewer who smoked exclusively responded by using e-cigarettes compared to those who dual used (3.9% vs. 43.7%). Quitting all tobacco use (ie, no smoking, vaping, or any tobacco use) was uncommon for both groups (3.6% vs. 9.0%). Under a hypothetical ban, majorities of those who exclusively smoke and who dual use predicted they would continue smoking (72.2% vs. 71.8%); fewer who smoke exclusively would use e-cigarettes compared to those who dual use (14.7% vs. 41.4%). Those who smoke exclusively were more likely to report quitting all tobacco compared to those who dual use (29.6% vs. 12.4%).

**Conclusions:**

Under real-world and hypothetical menthol cigarette bans, most respondents continued smoking. However, more young adults continued smoking following real-world bans, reflecting the limitations of local/state restrictions when menthol cigarettes are available in other jurisdictions.

**Implications:**

This survey asked young adults who use menthol cigarettes how they responded to real-world changes in the availability of menthol cigarettes; 89% reported continuing to smoke. Those who smoked exclusively were far less likely to respond by switching to e-cigarettes compared to people who dual used both products. Under a hypothetical federal menthol cigarette ban, 72% of young adults predicted that they would continue smoking. Quitting all tobacco was less common in the real-world scenario compared to the hypothetical ban. Access to menthol cigarettes in other jurisdictions and flavored cigars likely dampen the public health benefit of menthol cigarette bans.

## Introduction

The US Food and Drug Administration (FDA) plans to prohibit menthol flavoring in cigarettes.^1^ How young adults who smoke menthol cigarettes would respond is important to understand, as they are a priority population.^2^ To date, more than 170 localities and the state of Massachusetts have banned menthol flavored cigarettes.^3^ These policies provide an opportunity to analyze the impacts of a menthol cigarette ban prior to federal action. Menthol restrictions reduce health risks if people respond by quitting smoking or are dissuaded from initiating smoking, but their public health benefit is diminished if they continue smoking or using combustible tobacco. A menthol ban on cigarettes could also influence e-cigarette use because e-cigarettes and cigarettes are substitutes.^4–9^

A 2020 review summarized studies about real-world and hypothetical responses to menthol cigarette bans.^10^ Available real-world data come from other countries,^9,11^ and may not translate for the US context. Newer data about hypothetical menthol bans are needed because previous analyzes did not ask about switching to e-cigarettes, or because e-cigarettes available at the time differ substantially from those on the market today.^8,12–18^ Most surveys also fail to distinguish between responses from people who smoke exclusively and those who dual use with e-cigarettes, with exceptions.^19,20^ Respondents were typically allowed to select only one behavioral response to menthol bans. However, people who smoke may engage in multiple behaviors depending on the ease with which they could continue acquiring menthol cigarettes.

Recent surveys assessed potential responses to a comprehensive ban on all flavored combustible tobacco products (ban on both menthol cigarettes and flavored cigars).^20,21^ Although the FDA has proposed to ban menthol cigarettes and flavored cigars, these are being pursued as two separate product standards. Litigation could mean that only one of these product standards survives legal challenges, or more optimistically, that one would be implemented earlier than another.

Detailed, up-to-date estimates of menthol cigarette ban responses, both real-world and hypothetical, are needed to assess the potential impacts of a future federal menthol cigarette ban.

## Methods

An online, national survey of young adults ages 18‐34 who smoke menthol cigarettes (*N*  = 734) was conducted in 2021. Respondents in Massachusetts were oversampled to obtain sufficient real-world responses to a menthol ban. Participants were asked if at some point between Thanksgiving 2019 and the time of the survey, they were unable to buy menthol cigarettes—this was prior to the federal Tobacco 21 law (December 2019) and most state flavor bans (November 2019–present; Thanksgiving 2019 was used as an anchor to reduce recall error risk.) Those who responded affirmatively and smoked on ≥7 of 30 days before Thanksgiving 2019 *(n* = 416) received questions about real-world menthol bans; the remaining sample received questions about a hypothetical menthol ban and must have smoked on ≥7 of past 30 days (*n* = 318). Those who exclusively smoked reported no e-cigarette use in the past 30 days, while those who dual used had vaped on ≥1 of 30 days.

The sample was restricted to individuals who smoked menthol cigarettes, reported currently residing in the same state as they did on Thanksgiving 2019, and were unaffected by Tobacco 21 laws.

### Real-World Wenthol Cigarette Ban

We operationalized the real-world effect of a “ban” as the inability to purchase menthol cigarettes as usual. Those who stated that they could not purchase menthol cigarettes were asked, “After this change when you could not purchase menthol cigarettes, what did you do? Select all that apply.” Respondents who stated that they continued smoking menthol cigarettes were then asked: “How were you able to continue obtaining menthol cigarettes?” Response options included purchasing from the black market, from another jurisdiction [state, locality, or country], online, or obtaining through family and friends, modifying their cigarettes, or other.

### Hypothetical Menthol Cigarette Ban

Respondents who did not notice a change in their ability to purchase menthol cigarette as usual were asked: “Suppose the federal government implements a ban on sales of all menthol cigarettes in the United States and you are no longer able to purchase them through your usual source. What do you think you would do? Select all that apply.” Those who stated that they would continue using menthol cigarettes in response to a hypothetical ban were then asked: “How would you try to continue obtaining menthol cigarettes?”

For each outcome, we calculated the weighted proportion of individuals reporting each of the real-world or hypothetical ban outcomes. Individual responses were weighted by the inverse of the population prevalence of each survey quota group. Weighted and unweighted estimates were similar. Results by gender, race/ethnicity, and education were qualitatively similar (See Supplementary material).

## Results

Tobacco use responses to bans were categorized into four types: combustible tobacco use (C), heated or smokeless tobacco use (S), e-cigarette use (E), and quitting all forms of tobacco (Q). Responses were analyzed separately for people who exclusively smoke cigarettes versus people who dual use e-cigarettes and cigarettes. In both real-world and hypothetical menthol cigarette ban surveys, most people who exclusively smoke (95.8% and 81.4%), and the majority of people who dual use (59.7% and 70.4%) selected only one response option. Weighted responses are presented.

Table 1 shows responses to real-world menthol cigarette bans among young adults who smoke menthol cigarettes. The overwhelming majority—9 in 10 people—reported continuing to use combustible tobacco in some form. A substantial minority (43.7%) who dual use reported vaping after the ban versus 3.9% who exclusively smoked. Very few (1.5%) of those who exclusively smoke used heated or smokeless tobacco use versus 16.4% who dual use. People who dual use were more likely to include quitting all tobacco as a response compared to people who exclusively smoke (9.0% vs. 3.6%) but only < 2% of all respondents listed quitting as their sole response. The overwhelming majority of people who exclusively smoke (93%) listed continuing to smoke as their only response, compared to 48.2% who dual use.

**Table 1.** Responses to Real-World Menthol Cigarette ban Among US Young Adults who use Menthol Cigarettes

Young adults (18‐34) who smoke menthol cigarettes, *n* = 416		Responses to ban				Young adults who exclusively smoke cigarettes, *n* = 108			Young adults who dual use, *n* = 308		
		C	S	E	Q	*n*	%		*n*	%	
Any use of combustible tobacco, *n* = 361, 89.0%		√				98	93.0	*N* = 102, 95.3%	145	48.2	*N* = 259, 86.9%
		√	√			1	0.4		8	3.1	
		√		√		2	1.6		66	22.4	
		√	√	√		0	0		15	5.6	
		√		√	√	0	0		7	2.2	
		√	√		√	0	0		5	1.8	
		√	√	√	√	0	0		11	3.0	
		√			√	1	0.3		2	0.5	
Any use of heated or smokeless tobacco use, but not combustible tobacco, *n* = 10, 2.4%			√			1	1.1	*N* = 1, 1.1%	4	1.3	*N* = 9, 2.9%
			√	√		0	0		4	1.5	
			√		√	0	0		1	0.1	
			√	√	√	0	0		0	0	
Continue vaping only, or quit all tobacco, *n* = 39, 7.3%				√		1	0.4	*N* = 3, 2.3%	35	8.9	*N* = 36, 9%
				√	√	2	2.0		1	0.1	
Quit all tobacco, *n* = 6, 1.3%					√	2	1.3	*N* = 2, 1.3%	4	1.3	*N* = 4, 1.3%
Young adults who exclusively smoke cigarettes	*n* ^*^	102	2	5	5						
	%*	95.3	1.5	3.9	3.6						
Young adults who dual use	n*	259	48	139	31						
	%*	86.9	16.4	43.7	9.0						

Survey respondents who reported being affected by menthol cigarette restrictions were asked, “After this change when you could not purchase menthol cigarettes, what did you do? Select all that apply.” C = continued smoking menthol cigarettes by getting them from a different source, switch to non-menthol cigarettes or other combustible tobacco (eg. cigars, hookah, pipe tobacco, bidis); S = switch to using smokeless tobacco (eg, chewing tobacco, snus, snuff, dip, dissolvables) or heated tobacco (eg, IQOS, eclipse); E = switch to using flavored e-cigarettes; Q = quit all smoking or quit all tobacco use. Each row represents one of 15 possible response combinations: gray boxes with check marks represent the selected response for each product category, while dark gray boxes highlight respondents who only reported a single response type.

^*^Numbers in columns do not sum to 100% of the sample because categories are not mutually exclusive.

Table 2 shows responses to a hypothetical federal menthol cigarette ban among people who smoke menthol cigarettes. Two-thirds of respondents listed continued combustible tobacco use as one of their responses. Among people who dual use, e-cigarette use was the second most reported response option—endorsed by 41.4%—followed by quitting (12.4%). Among those who exclusively smoke, the reverse was true: the second most common response option was quitting (29.6%) followed by e-cigarette use (14.7%). Smokeless or heated tobacco use were least favored by both groups. Most respondents reported only one response type. A total of 56.7% who exclusively smoke stated that their only response would be to continue using combustible tobacco, whereas 44.8% who dual use did so. Those who exclusive smoke were more than twice as likely to report quitting all forms of tobacco as their only response than to report e-cigarette use as their only response (16.7% vs. 8.0%). This was reversed for those who dual use both products (7.8% vs. 15.8%).

**Table 2.** Responses to Hypothetical Menthol Cigarette ban Among US Young Adults who use Menthol Cigarettes.

Young adults (18‐34) who smoke menthol cigarettes, *n* = 318		Responses to ban				Young adults who exclusively smoke, *n* = 146			Young adults who dual use, *n* = 172		
		C	S	E	Q	n	%		n	%	
Any combustible tobacco use, *n* = 224, 72%		√				87	56.7	*N* = 106, 72.2%	68	44.8	*N* = 118, 71.8%
		√	√			4	0.9		4	2.2	
		√		√		6	3.6		33	18.4	
		√	√	√		0	0		7	2.9	
		√		√	√	0	0		3	2.0	
		√	√		√	0	0		0	0	
		√	√	√	√	0	0		1	0.7	
		√			√	9	11.0		2	0.8	
Any heated or smokeless tobacco use, but no combustible tobacco use, *n* = 10, 3.2%			√			0	0	*N* = 1, 1.2%	3	2.0	*N* = 9, 4.6%
			√	√		1	1.2		3	1.5	
			√		√	0	0		2	0.9	
			√	√	√	0	0		1	0.2	
Continue vaping only, or quit all tobacco, *n* = 44, 13.5%				√		11	8.0	*N* = 14, 9.9%	30	15.8	*N* = 30, 15.8%
				√	√	3	2.0		0	0	
Quit all tobacco, *n* = 40, 11.3%					√	25	16.7	*N* = 25, 16.7%	18	7.8	*N* = 15, 7.8%
Young adults who exclusively smoke cigarettes	n*	106	5	21	37						
	%*	72.2	2.1	14.7	29.6						
Young adults who dual use	n*	118	21	78	24						
	%*	71.8	10.3	41.4	12.4						

Survey respondents were asked, “Suppose the federal government implements a ban on sales of all menthol cigarettes in the United States and you are no longer able to purchase them through your usual source. What do you think you would do? Select all that apply.” C = continue smoking menthol cigarettes by getting them from a different source, switch to non-menthol cigarettes or other combustible tobacco (eg, cigars, hookah, pipe tobacco, bidis); S = switch to using smokeless tobacco (eg, chewing tobacco, snus, snuff, dip, dissolvables) or heated tobacco (eg, IQOS, eclipse); E = switch to using flavored e-cigarettes; Q = quit all smoking and tobacco use. Each row represents one of 15 possible response combinations: gray boxes with check marks represent the selected response for each product category, while dark gray boxes highlight respondents who only reported a single response type. *Numbers in columns do not sum to 100% of the sample because categories are not mutually exclusive.

Eight in 10 respondents who experienced real-world menthol cigarette bans stated that they purchased menthol cigarettes from another state, locality, or country (Supplementary Table S3). The second most common strategy was to obtain menthol cigarettes through family and friends (28.1%), followed by purchasing through illicit sources (13.8%). Few purchased menthol cigarettes online (7.7%) or modified their cigarettes (6.1%).

Among people who stated that they would continue smoking menthol cigarettes in the event of a hypothetical menthol ban, 54.2% would try to purchase them online, and 56.2% would access them through family and friends. 27.1 % would modify cigarettes by adding in menthol as a flavor, while 27.5% would purchase from another country and 24.5% through illicit market purchases.

## Discussion

This study assesses responses to real-world and hypothetical menthol cigarette bans among young adults who smoke menthol cigarettes. These estimates provide a range for the distribution of plausible behavioral responses to menthol bans among young adults who smoke menthol cigarettes.

Under real-world bans, 9 out of 10 people who use menthol cigarettes continued smoking combustible tobacco in some form. This can be explained by both their willingness to smoke non-menthol cigarettes, access to flavored cigars, as well as the availability of menthol flavored cigarettes beyond state or local borders. Still, if 10% of young adults quit smoking because of menthol bans, this would represent large health gains compared to other tobacco control interventions.^22^ Nearly half who dual use reported using e-cigarettes following bans, compared to 1 in 7 who exclusively smoke. Small proportions of respondents reported quitting all tobacco products or vaping exclusively, though this was more common among those who dual use. However, responses to local and state policies will differ from federal policy.

Under a hypothetical federal menthol ban, 72% of those who use menthol cigarettes predicted that they would continue to smoke; 31% would switch to e-cigarettes, and 19.1% would quit tobacco use altogether. In total, 56.7% who exclusively smoke would continue only smoking combustible tobacco rather than quit (16.7%) or switch to e-cigarettes (8%). Among those who exclusively smoke, the second most common response option was quitting tobacco products altogether (29.6%), followed by switching to e-cigarettes (14.7%). The reverse was true for those who dual use: more reported that they would switch completely to e-cigarettes (41.4%) than quit tobacco altogether (12.4%). Among young adults who dual use, twice as many would continue vaping compared to quitting as their only response option (15.8% vs. 7.8%).

Our results are consistent with Yang et al., which examined hypothetical responses to a ban on both menthol cigarettes and flavored cigars.^20^ Their study found that the majority of people who smoke menthol cigarettes would continue to smoke (53.6%), followed by switching to e-cigarettes (25.6%), and quitting all tobacco products (7.0%). Unlike Yang et al., we assess responses to a hypothetical menthol cigarette ban without a simultaneous flavored cigar ban. Our study indicates that higher proportions would continue to smoke combustible tobacco (young adults who exclusively smoke: 72.2%, dual use: 71.8%), that switching to e-cigarettes differs dramatically for people who smoke exclusive vs. dual use 14.7% vs. 41.4%, and more young adults who exclusively smoke than dual use would consider quitting all tobacco products (29.6% vs. 12.4%). Our results suggest that fewer young adults predict they would quit compared to a previous scoping review that estimated 25%‐64% would attempt to quit.^10^ Taken together, the results demonstrate the importance of a simultaneous flavored cigar ban to maximize reductions to smoking under a menthol cigarette ban.

## Strengths

This study more closely mimics real-world possibilities because participants could select multiple responses. Under real-world bans, young adults have multiple options available, including switching to other products, quitting all tobacco, and searching for alternative methods of acquiring menthol cigarettes. Unlike other studies, this survey directly asked consumers (1) whether they personally noticed the change in availability of menthol cigarettes—a better indication that a menthol ban affected them, (2) why they changed their behavior, and (3) whether that can be attributed directly to the absence of menthol cigarettes at retail stores.

## Limitations

This study is limited by participant samples that are not nationally representative, as Massachusetts was oversampled. Information about behaviors pre/post flavor bans were assessed retrospectively, which may introduce recall bias. Importantly, many local and state flavor bans coincided with COVID-19 disruptions.

Finally, our survey assessed responses to a menthol ban applied to cigarettes but not flavored cigars. Future studies will also need to separately analyze the potential effects of FDA flavor restrictions on combustible cigarettes and cigars, since one or both product standards could be interrupted by litigation. Research must also evaluate how responses to a menthol ban would change in the absence of flavored e-cigarettes—applications for which have yet to receive marketing authorization from FDA.

Menthol cigarette bans appear to encourage young adults who smoke away from cigarette use. This pattern was consistent between responses to real-world and hypothetical bans, though more pronounced in the latter. This suggests the limitations of state or local bans when users can access products in other jurisdictions or that people overestimate their ability to quit smoking under a hypothetical ban. A federal menthol cigarette ban would reduce smoking and would likely be strengthened by concurrent restrictions on flavored cigars.

## Supplementary Material

Supplementary material is available at *Nicotine and Tobacco Research* online.

ntad259_suppl_Supplementary_Material

## Data Availability

The data underlying this article will be made available upon request to the authors.
