# Supplementary material for: Responses to Real-World and Hypothetical Menthol Flavor Bans Among US Young Adults Who Smoke Menthol Cigarettes
Source: Nicotine Tob Res. 2023 Dec 26;26(6):785–9. doi: 10.1093/ntr/ntad259 (PMC11109486; doi:10.1093/ntr/ntad259)
Supplement: ntad259_suppl_Supplementary_Material [file ntad259_suppl_supplementary_material.docx]

**SUPPLEMENT**

**Survey design and sample recruitment**

Survey data were collected using Qualtrics from March 4, 2021 through December 23, 2021. Respondents could participate by taking the survey on a smartphone and/or computer; the majority used a smartphone. The survey was initially piloted on ~50 respondents before being launched more widely. To avoid idle sessions, respondents were also asked to move a slider on their screen before proceeding to the next session. A minimum time threshold was used to exclude respondents who rushed through their responses. Duplicate responses were also removed.

The overall survey was designed to capture adult responses to real-world and hypothetical bans applied to flavored e-cigarettes and menthol cigarettes. The present study only provides results for the latter set of survey questions focused on menthol cigarette bans. Given the wide range of tobacco control and flavor policy environments across states on Thanksgiving 2019, survey quotas were designed to obtain a more representative sample of smokers of ages 18-32 in each of three targeted state groups with distinct policy environments:

- Group 1 included states with existing bans on flavored e-cigarettes and tobacco 21 law (MA, NJ, NY, RI).
- Group 2 included states with state-enacted tobacco 21 laws but not flavor restrictions (HI,DC, CA, OR, ME, IL, ,VA, DE, AK, TX, VT, CT, MD, OH).
- Group 3 included states with neither flavor bans nor state-enacted tobacco-21 laws (AL, AK, AZ, FL, ID, KS, LS, MI, MO, MN, NV, NC, ND, SC, WV, WI, WA, OK, IA, IN, PA, SD, UT, WY, MS, CO, NH, GA, KT, MN, NE, NM, TN).

We aimed to recruit 1000 participants from state group 1, 500 from state group 2, and 500 from state group 3. Subgroup quotas in each state group were based on the corresponding prevalence of smoking by gender (men, women), age (18-22, 23-27 and 28-32), race (Black vs Non-Black) and education (high school degree or less vs. some college or more). Prevalence estimates used for determining the quotas were based on Tobacco Use Supplement to the Current Population Survey (TUS-CPS) 2018-2019 data.

Due to an insufficient number of participants who smoked cigarettes in the state of Massachusetts, the only state with a real-world menthol cigarette ban, the age limit was relaxed to include adults between ages 33-41, although the present analysis is restricted to young adults ages 18-34.

The final samples sizes per state group were 523 individuals from group 1, 77 individuals from group 2, and 134 individuals from group 3.

The study was approved by the Yale Human Subjects Committee and University of Michigan Institutional Review Board Health Sciences and Behavioral Sciences (HUM #00190165) and deemed exempt from continued oversight.

**Inclusion criteria**

Figure S1 presents a flowchart of the sample inclusion criteria. Respondents were included in this survey if they “had smoked at least 100 cigarettes in their lifetime” and “reported smoking on ≥7 days in the 30 days before Thanksgiving 2019”. The sample was restricted to people who lived in the same state at the time of survey as they did on Thanksgiving 2019. Of the 734 total respondents, 38 of them were individuals who dual use cigarettes and e-cigarettes and were recruited through the “Vaping Survey”, similar survey focused on e-cigarette use and flavor bans. These individuals were asked the same set of questions but were asked questions about e-cigarette use first before questions about cigarette smoking.

Participants were asked “Do you ever remember a time when you first found that you could not buy menthol cigarettes as usual”? Those who responded “Yes, I remember” were assigned the Real-World Menthol Ban questions. Those who responded “No, I do not remember” were assigned to the Hypothetical Federal Flavor Ban questions.

Because some individuals may have been unable to purchase menthol cigarettes due to Tobacco 21 laws, we asked respondents to select reasons why they were unable to purchase them. Those who stated “I was under age 21 at the time” were excluded from analyses. Only those who selected “Menthol cigarettes were not available at retail stores” as their reason were considered to have been affected by real-world menthol bans.

Note that Question 81 erroneously excluded an option to select ‘Switch to using tobacco-flavored e-cigarettes’.

**Subgroup analyses**

Additional disaggregated results with 95% confidence intervals by gender (men, women), race (Black NH, White NH, Hispanic), age (18-34, 35-41), and education (college degree or more, less than college degree) are available in the supplemental Excel file. Small sample sizes make some subgroup estimates unreliable. Hypothetical ban data for ages 35-41 are excluded due to insufficient sample (n=20).

All analyses were conducted in R.

**Figure S1. Menthol cigarette flavor ban survey flowchart and sample inclusion criteria**

Do you ever remember a time when you first found that you could not buy menthol cigarettes as usual?

No, I do not smoke menthol cigarettes

N = 205

Dual use on Thanksgiving 2019

N = 1187

**Hypothetical Menthol Ban**

N = 318

**Real-World
Menthol Ban**

N = 416

During the time when you were unable to purchase menthol cigarettes, why were you unable to purchase them?*

I was under age 21 at the time,

I don’t know,

Something else

N = 365

**Exclusions**

Exclusive smoking on Thanksgiving 2019

N = 443

In the 30 days before Thanksgiving 2019, on how many days do you think you smoked cigarettes? (0-30)

Have you smoked at least 100 cigarettes in your entire life?

Which state do you currently live in now?

Did you live in this same state on Thanksgiving 2019?

I do not currently reside in the U.S.

No

No,

Don’t know

<7 days

<7 days

N=326

**Smoking Survey**

N = 1521

**Vaping Survey**

N = 109

In the 30 days before Thanksgiving 2019, on how many days do you think you used e-cigarettes? (0-30)

On how many of the past 30 days have you smoked cigarettes?

*Respondents were prompted to select all that apply. Responses that did not include “Menthol cigarettes were not available at retail stores” were excluded. Any responses that included “I was under 21 at the time” were excluded.

**Table S1. Real-world menthol cigarette flavor ban survey, sample characteristics**

| **Real world menthol flavor ban survey** | **Exclusive cigarette smoking  on Thanksgiving 2019** | | **Dual use  on Thanksgiving 2019** | |
| --- | --- | --- | --- | --- |
|  | **n= 108** | **weighted %** | **n= 308** | **weighted %** |
|  |  |  |  |  |
| **Age** |  |  |  |  |
| 18-21 | 5 | 13.3% | 19 | 10.1% |
| 22-24 | 8 | 14.9% | 44 | 13.9% |
| 25-29 | 47 | 36.1% | 104 | 30.6% |
| 30-34 | 48 | 35.6% | 141 | 45.5% |
| **Gender** |  |  |  |  |
| Man | 35 | 39.7% | 153 | 48.7% |
| Woman | 70 | 57.7% | 146 | 46.8% |
| Non-binary / Prefer not to say | 3 | 2.6% | 9 | 4.5% |
| **Race/Hispanic origin** |  |  |  |  |
| Hispanic | 18 | 28.0% | 72 | 20.7% |
| Black, non-Hispanic | 15 | 12.9% | 18 | 7.4% |
| White, non-Hispanic | 72 | 57.3% | 206 | 68.1% |
| Other, non-Hispanic | 3 | 1.8% | 12 | 3.8% |
| **Education** |  |  |  |  |
| High school degree or Less | 61 | 45.5% | 94 | 23.0% |
| Some college | 38 | 43.5% | 84 | 32.8% |
| At least college degree | 9 | 10.9% | 130 | 44.2% |
| **Census region** |  |  |  |  |
| Northeast | 98 | 94.2% | 250 | 88.2% |
| Midwest | 2 | 1.1% | 11 | 2.1% |
| South | 3 | 1.7% | 12 | 2.6% |
| West | 5 | 3.0% | 35 | 7.1% |
| **State of Massachusetts** |  |  |  |  |
| Yes | 87 | 81.1% | 162 | 55.4% |
| No | 21 | 18.9% | 146 | 44.6% |
| **E-cigarette use in 30 days pre-Thanksgiving 2019** |  |  |  |  |
| 0 days | 108 | 100% | 0 | 0% |
| 1-6 days | 0 | 0% | 73 | 20.6% |
| 7-10 days | 0 | 0% | 37 | 11.4% |
| 11-20 days | 0 | 0% | 86 | 26.3% |
| 21-30 days | 0 | 0% | 112 | 41.7% |
| **Cigarette smoking in 30 days pre-Thanksgiving 2019** |  |  |  |  |
| 0 days | 0 | 0% | 0 | 0% |
| 1-6 days | 0 | 0% | 0 | 0% |
| 7-10 days | 7 | 4.2% | 42 | 13.1% |
| 11-20 days | 8 | 9.2% | 79 | 27.9% |
| 21-30 days | 93 | 86.6% | 187 | 59.0% |

**Table S2. Hypothetical federal menthol cigarette flavor ban survey, sample characteristics**

| **Hypothetical federal menthol flavor ban survey** | **Exclusive cigarette smoking*** | | **Dual use*** | |
| --- | --- | --- | --- | --- |
|  | **n= 146** | **weighted %** | **N= 172** | **weighted %** |
|  |  |  |  |  |
| **Age** |  |  |  |  |
| 18-21 | 10 | 16.1% | 17 | 11.4% |
| 22-24 | 25 | 20.6% | 27 | 15.5% |
| 25-29 | 56 | 34.0% | 74 | 42.2% |
| 30-34 | 55 | 29.3% | 54 | 30.9% |
| **Gender** |  |  |  |  |
| Man | 55 | 31.6% | 85 | 38.0% |
| Woman | 82 | 58.6% | 79 | 55.3% |
| Non-binary / Prefer not to say | 9 | 9.8% | 8 | 6.6 |
| **Race/Hispanic origin** |  |  |  |  |
| Hispanic | 18 | 14.7% | 33 | 17.7% |
| Black, non-Hispanic | 28 | 24.8% | 19 | 13.8% |
| White, non-Hispanic | 89 | 55.6% | 107 | 62.2% |
| Other, non-Hispanic | 11 | 4.8% | 13 | 6.4% |
| **Education** |  |  |  |  |
| High school degree or Less | 82 | 41.2% | 74 | 28.1% |
| Some college | 41 | 39.7% | 49 | 37.4% |
| At least college degree | 23 | 19.0% | 49 | 34.5% |
| **Census region** |  |  |  |  |
| Northeast | 91 | 62.5% | 104 | 75.2 |
| Midwest | 16 | 10.7% | 17 | 5.6 |
| South | 17 | 9.2% | 17 | 6.6 |
| West | 22 | 17.5% | 34 | 12.6 |
| **State of Massachusetts** |  |  |  |  |
| Yes | 14 | 15.7% | 25 | 16.4% |
| No | 132 | 84.3 | 147 | 83.6% |
| **E-cigarette use in past 30 days*** |  |  |  |  |
| 0 days | 146 | 100% | 0 | 0% |
| 1-6 days | 0 | 0% | 29 | 19.2% |
| 7-10 days | 0 | 0% | 28 | 15.1% |
| 11-20 days | 0 | 0% | 57 | 33.8% |
| 21-30 days | 0 | 0% | 58 | 31.8% |
| **Cigarette smoking in past 30 days*** |  |  |  |  |
| 0 days | 0 | 0% | 0 | 0% |
| 1-6 days | 0 | 0% | 0 | 0% |
| 7-10 days | 10 | 6.4% | 19 | 11.3% |
| 11-20 days | 20 | 23.3% | 39 | 29.3% |
| 21-30 days | 116 | 70.3% | 114 | 59.5% |

Note: These survey questions reflect tobacco use at time of survey, and not on Thanksgiving 2019: “On how many of the past 30 days have you used e-cigarettes?”, “On how many of the past 30 days have you smoked cigarettes?”

*Real-World Menthol Ban*

Q68 Do you ever remember a time when you first found that you could not buy **menthol cigarettes** as usual?

- Yes, I remember
- No, I do not remember
- No, I do not smoke menthol cigarettes.

Q72 During the time when you were unable to purchase menthol cigarettes, why were you unable to purchase them? Select all that apply.

- Menthol cigarettes were not available at retail stores
- I was under age 21 at the time
- Something else ________________________________________________
- I don't know

Q73 After this change when you could not purchase menthol cigarettes, what did you do? Select all that apply.

- Switch to non-menthol cigarettes
- Switch to smoking other combustible tobacco (e.g. cigars, hookah, pipe tobacco, bidis)
- Switch to using flavored e-cigarettes
- Switch to using tobacco-flavored e-cigarettes
- Switch to using smokeless tobacco (e.g. chewing tobacco, snus, snuff, dip, dissolvables)
- Switch to using heated tobacco (e.g. IQOS, Eclipse)
- Quit all smoking
- Quit all tobacco product use
- Continued smoking menthol cigarettes by getting them from a different source
- Continued smoking by buying menthol cigarettes with a fake ID

[If “Continue smoking menthol cigarettes by getting them from a difference source”]

Q74 How were you able to continue obtaining menthol cigarettes? Select all that apply.

- Purchased from the black market
- Purchased from another state, locality, or country
- Purchased online
- Obtained through family and friends
- Modified cigarettes on my own by adding menthol flavor
- Other

Q79 After you noticed you could not buy menthol cigarettes as usual, did this change your perceptions of the health risks of cigarettes overall?

- Yes, it seemed more risky to smoke cigarettes than before
- Yes, it seemed less risky to smoke cigarettes than before
- No

Q80 After you noticed that you could not buy as usual, did this change your perceptions of the health risks of smoking menthol cigarettes in particular?

- Yes, it seemed more risky to smoke menthol cigarettes as compared to non-menthol
- Yes, it seemed less risky to smoke menthol cigarettes as compared to non-menthol
- No

*Hypothetical Menthol Ban Scenario*

Q81 Suppose the federal government implements a ban on sales of all **menthol** **cigarettes** in the U.S. and you are no longer able to purchase them through your usual source. Select all that apply.
 
What do you think you would do?

- Switch to non-menthol cigarettes
- Switch to using combustible tobacco (e.g. cigars, hookah, pipe tobacco, bidis)
- Switch to using flavored e-cigarettes
- Switch to using smokeless tobacco (e.g. chewing tobacco, snus, snuff, dip, dissolvables)
- Switch to using heated tobacco (e.g. IQOS, Eclipse)
- Quit all smoking and tobacco use
- Continue smoking menthol cigarettes by getting them from a different source
- Nothing, I do not smoke menthol cigarettes
- Don’t know

[If “Continue smoking menthol cigarettes by getting them from a difference source”]

Q82 How would you try to continue obtaining menthol cigarettes?

- Purchase from another country
- Purchase online
- Get through family and friends
- Purchase from the black market
- Modify cigarettes on my own by adding menthol flavor
- Other (please specify) ________________________________________________
- Don’t know

**Table S3. Continued menthol cigarette smoking following a ban among US young adults 18-34 who use menthol cigarettes**

| Real world menthol cigarette ban | | | Hypothetical federal menthol cigarette ban | | |
| --- | --- | --- | --- | --- | --- |
| How were you able to continue obtaining menthol cigarettes? Select all that apply. | n | % | How would you try to continue obtaining menthol cigarettes? Select all that apply. | n | % |
| Purchased from the black market | 24 | 13.8  (7.9-19.7) | Purchase from the black market | 29 | 24.5  (13.8-35.2) |
| Purchased from another state, locality, or country | 134 | 79.8  (73.5-86.2) | Purchase from another country | 30 | 27.5  (16.3-38.8) |
| Purchased online | 14 | 7.7  (3.3-12) | Purchase online | 60 | 54.2  (40.6-67.9) |
| Obtained through family and friends | 58 | 28.1  (20.4-35.8) | Get through family and friends | 58 | 56.2  (43.2-69.2) |
| Modified cigarettes on my own by adding menthol flavor | 14 | 6.1  (2.4-9.8) | Modify cigarettes on my own by adding menthol flavor | 33 | 27.1  (16.5-37.7) |
| Other | 12 | 6.4  (2.2-10.5) | Other | 7 | 4.6  (0.5-8.7) |

Notes: Adults who use menthol cigarettes reported that they would ‘continue smoking menthol cigarettes by getting them from a different source’, following a hypothetical federal menthol cigarette ban (n= 111) or who reported that they ‘continued smoking menthol cigarettes by getting them from a different source’, following real world menthol cigarette bans (n=180). Numbers do not sum to 100% because respondents were allowed to select more than one option.
